# The Application of Quantitative ^1^H-NMR for the Determination of Melatonin and Vitamin B6 in Commercial Melatonin Products

**DOI:** 10.3390/molecules30142942

**Published:** 2025-07-11

**Authors:** Xinyu Gao, Jiahao Niu, Zhengjian Xiao, Da Rong, Mingming Yu, Sherwin K. B. Sy, Cong Wang, Zhihua Lv

**Affiliations:** 1 School of Medicine and Pharmacy, Ocean University of China, Qingdao 266003, China; gaoxinyu1332@stu.ouc.edu.cn (X.G.); niu199606@163.com (J.N.); xiaozhengjian180@163.com (Z.X.); 18363020623@163.com (D.R.); yumingming@ouc.edu.cn (M.Y.); 2 Laboratory for Marine Drugs and Bioproducts of Qingdao National Laboratory for Marine Science and Technology, Qingdao 266003, China; 3 Department of Statistics, State University of Maringá, Maringá 87020-900, Brazil; sherwin.kenneth.sy@gmail.com; 4 Key Laboratory of Glycoscience and Glycotechnology of Shandong Province, Qingdao 266003, China; 5 Key Laboratory of Marine Drugs, Ministry of Education of China, Qingdao 266003, China

**Keywords:** quantitative analysis, ^1^H-NMR, melatonin, vitamin B6, quality control, method validation

## Abstract

Melatonin supplements have been widely used to improve sleep quality and overcome sleep disorders, with melatonin and vitamin B6 serving as the primary active ingredients. This study developed a novel analytical method for the simultaneous quantification of melatonin and vitamin B6 using ^1^H-NMR spectroscopy. The characteristic signals of melatonin and vitamin B6 hydrochloride at δ 7.09 ppm and δ 8.12 ppm were selected for quantitative analysis, with maleic acid used as the internal standard. The method was validated for specificity, precision, and stability. The results demonstrate that the method exhibits high precision and complies with the guidelines established by the China Food and Drug Administration (CFDA). Furthermore, this method has been successfully applied to commercially available formulations. Compared to conventional methods, the ^1^H-NMR technique offers a more efficient and simpler alternative, making it suitable for the simultaneous quantitative determination of melatonin and vitamin B6 hydrochloride. This approach ensures the quality, stability, and safety of commercial melatonin products.

## 1. Introduction

Sleep constitutes one-third of human life, and maintaining good sleep habits is essential for improving quality of life and preventing health problems. However, with societal developments, sleep disorders have become increasingly prevalent, seriously affecting both the physical and mental health of people across all ages [1,2]. Common sleep disorders include insomnia, circadian rhythm disorders, sleep-disordered breathing, hypersomnia/narcolepsy, parasomnias, and restless leg syndrome/periodic limb movement disorder [3]. Treatments for sleep disorders include medication and cognitive behavioral therapy, with medication being one of the most commonly used treatments.

**Figure 1:**
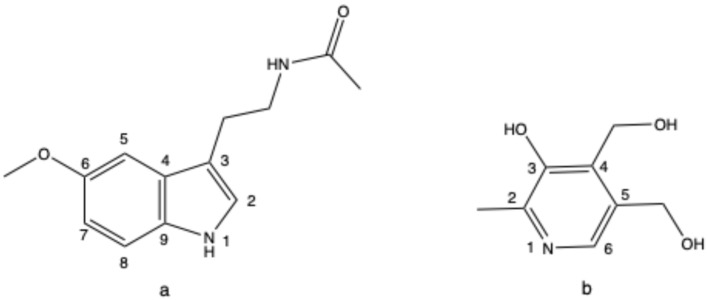
The structures of (**a**) melatonin and (**b**) vitamin B6.

Melatonin, a hormone produced by the pineal gland, is present and detectable in different sources, including honey [4]. Disruptions in its secretion may lead to insomnia [5]. The hypnotic effect of melatonin is well-documented in the literature, and it is associated with fewer side effects compared to other medications, making it a commonly used medication for treating sleep disorders [6,7,8]. Vitamin B6, which cannot be synthesized by the human body, must be obtained from dietary sources such as fruits and vegetables [9]. A deficiency of vitamin B6 can lead to symptoms such as anemia, neurological disorders, and skin disorders, making the adequate intake of vitamin B6 essential for good health [10]. However, the prolonged excessive intake of vitamin B6 may cause neurological damage, though this condition typically resolves after discontinuing its use [11]. Vitamin B6 plays a crucial role as a coenzyme in the biosynthesis of melatonin, and its deficiency has been associated with sleep disorders [12]. The combination of melatonin and vitamin B6 (chemical structures shown in Figure 1) is commonly used to improve sleep disorders.

The accurate measurement of active ingredients in nutraceuticals is essential to ensure both the safety and efficacy of products. For melatonin supplements, an excessively high dose may lead to drowsiness or neurological problems, while an inadequate dose may fail to improve sleep quality; the quality control of melatonin and vitamin B6 is crucial. Several analytical techniques, such as gas chromatography–mass spectrometry (GC-MS) [13], high-performance liquid chromatography with fluorescence detection (HPLC-FLD) [14], and voltammetry [15], have been used to quantify melatonin and vitamin B6 in nutraceuticals. However, these methods may be less efficient in high-throughput analysis due to requirements such as mobile phase preparation, column equilibration, electrode pretreatment, and the need for separate calibration curves. In contrast, ^1^H-NMR provides a faster, simultaneous quantitative analysis solution. Therefore, a rapid and efficient method for the simultaneous quantification of melatonin and vitamin B6 is needed.

Nuclear magnetic resonance (NMR) is a non-destructive analytical technique that utilizes a magnetic field for the qualitative and quantitative analysis of samples. With advancements in NMR instrumentation and techniques, ^1^H-NMR has gained widespread application in food science [16,17,18], herbal medicine identification [19,20], pharmaceutical quality control [21,22,23], metabolomics studies [24,25,26], and material characterization [27]. In quantitative analysis, NMR enables both qualitative and quantitative results and allows the direct analysis of mixtures without the need for calibration curves, pre-separation, or purification [28]. Importantly, the quantification process does not rely on analytical standards but instead uses any high-purity substance as an internal standard [29,30,31]. The International Conference on Harmonization (ICH) [32], European Pharmacopoeia, the United States Pharmacopoeia, and Chinese Pharmacopoeia have included ^1^H-NMR methods to meet new quality control standards.

The quantification of melatonin and vitamin B6 by ^1^H-NMR offers a more efficient and reliable alternative method for the quality control of melatonin supplements. In this study, we developed a rapid and accurate ^1^H-NMR method for the simultaneous quantification of melatonin and vitamin B6 hydrochloride in commercial melatonin products. To validate the reliability of the method, we compared it with a conventional high-performance liquid chromatography (HPLC) method.

## 2. Results and Discussion

### 2.1. ^1^H-NMR Method Development

**Figure 2:**
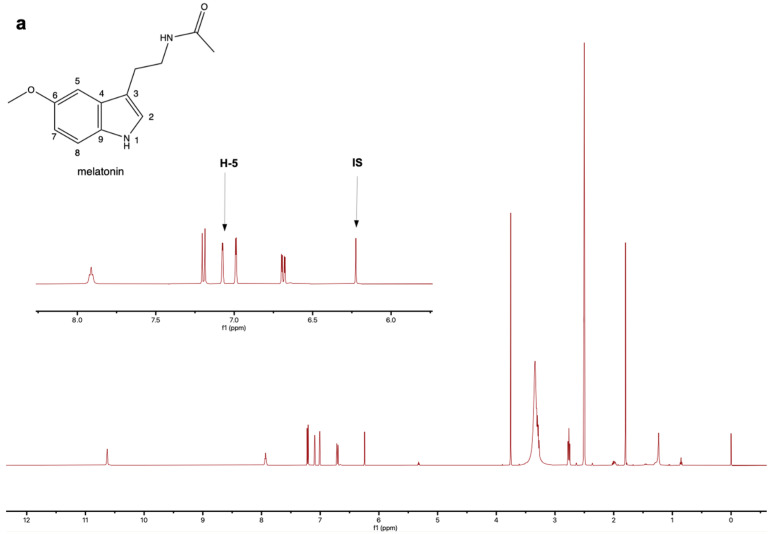

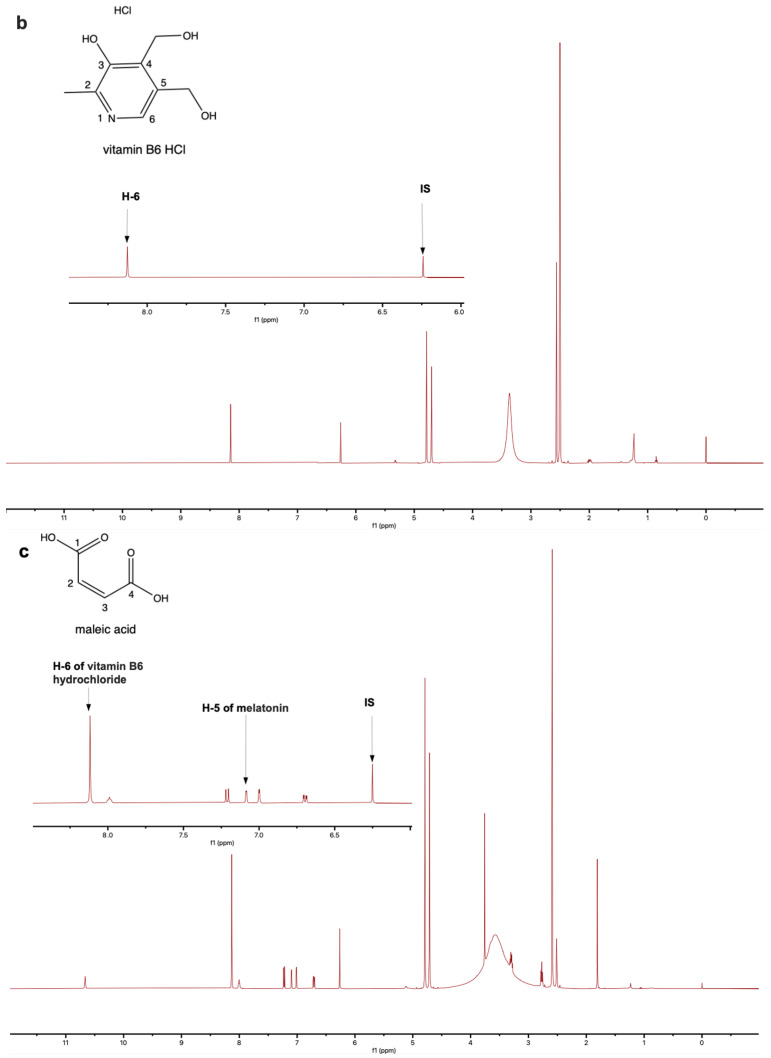
^1^H-NMR spectra (DMSO-d6, 500 MHz) of (**a**) the melatonin standard (pure reference compound), (**b**) the vitamin B6 hydrochloride standard, and (**c**) the melatonin sample (commercial product). IS: internal standard, maleic acid.

DMSO-d6 was chosen as the solvent because melatonin and vitamin B6 hydrochloride exhibit better solubility in DMSO-d6. The ^1^H-NMR spectra of melatonin and vitamin B6 hydrochloride standards, as well as melatonin samples, are shown in Figure 2. The signal of maleic acid at δ 6.27 ppm showed no overlap with the signals of melatonin and vitamin B6 hydrochloride, and thus, we selected this as the quantitative peak for the internal standard. In this study, the signals of melatonin and vitamin B6 hydrochloride at δ 7.09 ppm and δ 8.12 ppm were chosen for quantification. These peaks exhibited good resolution (Figure 2) and showed no significant overlap with signals from other components in the melatonin sample. Therefore, these peaks could be reliably used for the simultaneous quantification of melatonin and vitamin B6 hydrochloride.

In ^1^H-NMR analysis, the relaxation delay (D1) is an important parameter affecting the accuracy of quantitative measurements [33]. To assess its impact, we optimized D1 by testing values at 1, 2, 5, 10, 15, 20, and 25 s. The results showed that when D1 ≤ 10 s, the ratio $A_{X}/A_{IS}$ gradually increased with increasing D1 and then stabilized. As a result, D1 was set to 10 s for subsequent quantitative analysis in this study.

We tested different pulse angles (30°, 45°, and 90°) under fixed conditions of D1 = 10 s and number of scans (NS) = 32. The results showed similar measurements across the pulse angles, with a relative standard deviation (RSD) of less than 2%, indicating that the pulse angle does not significantly affect the results. With D1 fixed at 10 s and the pulse angle fixed at 45°, we varied NS (1, 4, 8, 16, and 32) while increasing the sample concentration for sufficient signal intensity at lower NS values. The results showed similar measurements, with an RSD of less than 2%. However, the signal-to-noise ratio decreased as NS was reduced. Based on these results, we selected the conventional NS of 32 for quantitative analysis.

The final NMR acquisition parameters were as follows: pulse sequence s2pul; pulse angle 45°; NS 32; spectral width (SW) 8012.8 Hz; D1 10 s; and probe temperature 298 K.

### 2.2. Method Validation

**Figure 3:**
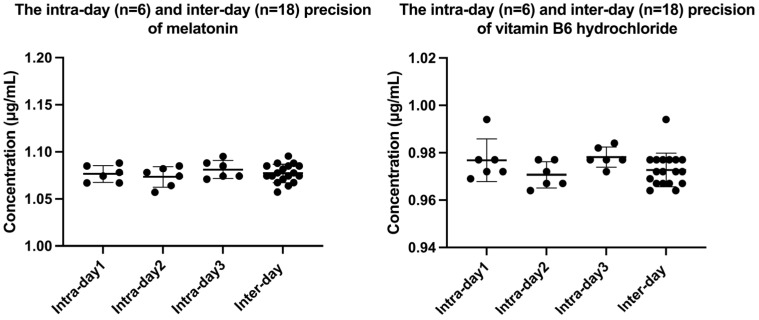
The intra-day (n = 6) and inter-day (n = 18, 3 days) precision of melatonin and vitamin B6 hydrochloride.

The ^1^H-NMR method showed excellent linearity for melatonin and vitamin B6 hydrochloride in the concentration ranges of 0.1–4 mg/mL and 0.1–5 mg/mL, respectively. The key validation parameters are summarized in Table 1 and Figure 3. Stability experiments confirmed that melatonin and vitamin B6 hydrochloride were stable within 72 h. The validation of the ^1^H-NMR method demonstrated that it met CFDA guidelines for accuracy and reliability. Additionally, the HPLC method also exhibited good linearity in the concentration range of 0.02–1 mg/mL. The linear regression equation for melatonin was y = 14,453x + 77,651 (R^2^ = 0.9995) and the range for vitamin B6 hydrochloride was y = 10,469x + 13,686 (R^2^ = 1) (Table 1).

### 2.3. Method Application

The ^1^H-NMR method was developed for the determination of melatonin and vitamin B6 hydrochloride in commercially available melatonin capsules. The results showed that the contents of melatonin and vitamin B6 hydrochloride were 105.8 ± 0.4 μg/10 mg and 368.0 ± 2.0 μg/10 mg, respectively (Table 2). To verify the accuracy of the ^1^H-NMR method, the same batch of samples was analyzed using HPLC, yielding results of 99.5 ± 0.2 μg/10 mg for melatonin and 365.2 ± 0.9 μg/10 mg for vitamin B6 hydrochloride (Table 2). The results from both methods were in good agreement, demonstrating that the ^1^H-NMR method is reliable for the quantification of these compounds in capsules. In conclusion, the ^1^H-NMR method developed is a rapid and accurate quantitative technique, making it a valuable supplement to traditional HPLC methods.

## 3. Materials and Methods

### 3.1. Chemicals and Materials

Melatonin (98%) and vitamin B6 hydrochloride (98%) standards were purchased from Aladdin (Shanghai, China). The maleic acid (internal standard, 98%) standard was purchased from Yuan Ye (Shanghai, China). Dimethyl sulfoxide-d6 (DMSO-d6, 99.9%) was obtained from Cambridge Isotope Laboratories, Inc. (Andover, MA, USA). All other solvents (HPLC grade) were purchased from Honeywell Burdick & Jackson Research Chemicals (Ulsan, Republic of Korea). Commercially available “melatonin” capsules were purchased from common health food brands.

### 3.2. Sample Preparation

A total of 5 mg of the maleic acid standard was added to DMSO-d6 as the solvent used to prepare a 1 mg/mL maleic acid stock solution in a 5 mL volumetric flask. This stock solution was further diluted with DMSO-d6 to prepare a 0.1 mg/mL maleic acid internal standard working solution. Similarly, 10 mg of melatonin and 25 mg of vitamin B6 hydrochloride standards were dissolved in the prepared internal standard working solution to obtain 2 mg/mL and 5 mg/mL stock solutions, respectively. Other concentrations were prepared by further dilution. The weighing accuracy of the sample was ±0.2 mg, and the precision was ≤0.1 mg. Calibration curves for melatonin and vitamin B6 hydrochloride were prepared in the concentration ranges of 0.1–4 mg/mL (0.1, 0.2, 0.5, 1, 2, and 4 mg/mL) and 0.1–5 mg/mL (0.1, 0.25, 0.5, 1, 2.5 and 5 mg/mL), respectively. The calibration curves were prepared fresh daily.

To extract the contents of the capsules, 10 mg of the capsule contents was mixed with 1 mL of an internal standard working solution. The mixture was vortexed for 1 min, then centrifuged at 14,000 rpm for 3 min at room temperature. In total, 500 μL of the supernatant was used for ^1^H-NMR analysis. For HPLC analysis, calibration curves for melatonin and vitamin B6 hydrochloride were constructed using eight concentrations (0.02, 0.05, 0.1, 0.2, 0.4, 0.5, 0.8, and 1 mg/mL) in the range of 0.02–1 mg/mL. In total, 10 mg of the capsule contents was weighed and dissolved in 1 mL of methanol–water (*v*/*v* = 60:40) solution. The mixture was vortexed for 1 min and then centrifuged at 14,000 rpm for 3 min at room temperature. The supernatant was used for the HPLC assay.

### 3.3. ^1^H-NMR Measurements

All ^1^H-NMR spectra were recorded using an Agilent DD2 500 MHz spectrometer equipped with a 5 mm One NMR probe (Santa Clara, CA, USA). For the ^1^H-NMR analysis, 500 μL of the supernatant was used as the sample volume. Prior to data acquisition, D1 was optimized by testing values of 1, 2, 5, 10, 15, 20, and 25 s to ensure complete spin-lattice relaxation.

In addition, experiments were conducted to evaluate the effects of pulse angles and NS on the NMR spectra. The pulse angles tested were 30°, 45°, and 90°, and the NS varied from 1, 4, 8, 16, to 32. The impact of these parameters on the quantitative results was analyzed to optimize the measurement conditions for accurate and reliable data.

Tetramethylsilane (TMS) was used as the internal reference standard for the ppm scale. All quantitative peaks used for ^1^H-NMR were phase- and baseline-corrected and integrated using MestReNova (v14.0.0, Mestrelab Research, Santiago, Spain).

### 3.4. HPLC-PDA Analysis

HPLC was employed to verify the accuracy of the ^1^H-NMR method. The analysis was conducted using a Waters e2695 system equipped with a 2998 PDA detector (Milford, MA, USA). A SHIMADZU (Tokyo, Japan) Shim-pack GIST C-18 column (5 μm, 4.6 mm × 150 mm) was used for the column separation of melatonin and vitamin B6 hydrochloride. The chromatographic conditions were as follows: column temperature at 30 °C; flow rate of 1 mL/min; injection volume of 10 μL; mobile phase of methanol–water (60∶40; *v*/*v*); isocratic elution; and a total sample run time of 10 min with a detection wavelength of 278 nm.

### 3.5. Identification of Contents

The quantification principle in ^1^H-NMR spectroscopy is based on the fact that the intensity of the signal in the NMR spectrum is proportional to the number of protons contributing to that resonance [34]. The calculation is as follows:

(1) $$W_{X}=W_{IS}\times \frac{A_{X}}{A_{IS}}\times \frac{N_{IS}}{N_{X}}\times \frac{M_{X}}{M_{IS}}\times P_{IS}$$

where W, A, N, and M represent the mass, integrated area, number of nuclei, and molar mass of the compound to be tested (X) and the internal standard (IS), respectively. P_IS_ represents the purity of IS. Each sample was integrated six times, and the average was taken to minimize errors. The signals of melatonin at 7.09 ppm (H-4), vitamin B6 hydrochloride at 8.12 ppm (H-6), and maleic acid at 6.27 ppm (H-2 and H-3, two protons) were used for quantitative analysis.

### 3.6. Quantitative ^1^H-NMR Validation Method

The validation test was conducted in accordance with the guidelines for the validation of chemical analytical methods set out in the Chinese Pharmacopoeia (2020 edition, Part IV) [35]. The LOD and LOQ were determined using signal-to-noise (S/N) values of 3 and 10 to ensure that the detected signal was statistically significant above noise levels and guarantee sufficient precision for quantification, respectively [36]. Specifically, the S/N value for the quantitative peaks was calculated using the dsnmax (200) code within the Vnmrj software (v3.2), which is provided with the Agilent DD2 500 MHz NMR spectrometer. Linearity was evaluated using at least 5 concentration points to construct the standard curve, with the correlation coefficient (r) typically greater than 0.99. Precision and reproducibility were determined with RSD values that did not exceed 6%. Stability was assessed by analyzing samples stored at room temperature for 0, 2, 4, 24, 48, and 72 h, with RSD values required to be ≤2%. The system suitability test was performed with six replicate scans of standard solutions. Recovery was evaluated with the spiking method, and the recovery rate was found to fall within the 80–120% range. The recovery was calculated according to the following equation:

(2) $$R=\frac{S_{1}-S_{0}}{S}\times 100\%$$

where *R* is the recovery and *S*_0_ and *S*_1_ represent the measured concentrations (mg/mL) of the analyte in the sample before and after the addition of a known amount of standard solution, respectively. *S* is the concentration (mg/mL) of the standard solution added to the sample. These values were measured using the ^1^H-NMR method.

## 4. Conclusions

In this study, an accurate ^1^H-NMR analytical method was developed for the simultaneous determination of melatonin and vitamin B6 hydrochloride levels in nutraceuticals. The method was validated and successfully applied to the quality control of commercially available products, demonstrating good reproducibility and reliability. Compared to traditional HPLC methods, the ^1^H-NMR method offers several advantages: it does not require an individual standard for each analyte, uses an internal standard for multi-component analysis, is easier to operate, and provides faster detection. Thus, it serves as an effective supplement to existing pharmacopeia methods. This study presents a feasible and efficient method for the quality control of melatonin and vitamin B6 hydrochloride in melatonin nutraceuticals.

## Figures and Tables

**Table 1:** The linear equation, linear range, LOD, LOQ, precision, reproducibility, and recovery of melatonin and vitamin B6 hydrochloride.

Method	Parameters		Melatonin	Vitamin B6 Hydrochloride
^1^H-NMR	Linear equation		y = 2.4839x − 0.0666	y = 2.8872x − 0.1193
	R squared		0.9985	0.9995
	Linear range (mg/mL)		0.1–4	0.1–5
	LOD (mg/mL)		0.007	0.006
	LOQ (mg/mL)		0.1	0.02
	Precision (RSD, %)	Intra-day	0.80–1.00	0.44–0.93
		Inter-day	0.90	0.73
	Reproducibility (%)		1.51	0.93
	Recovery (%)		107.3	97.8
HPLC	Linear equation		y = 14,453x + 77,651	y = 10,469x + 13,686
	R squared		0.9995	1.0000
	Linear range (mg/mL)		0.02–1	0.02–1

LOD, limit of detection; LOQ, limit of quantitation; N = 6.

**Table 2:** The amount (μg/10 mg) of melatonin and vitamin B6 hydrochloride in products.

Weight (mg)	Melatonin (μg)		Vitamin B6 Hydrochloride (μg)	
	^1^H-NMR	HPLC	^1^H-NMR	HPLC
10	105.8 ± 0.4	99.5 ± 0.2	368.0 ± 2.0	365.2 ± 0.9

N = 6.

## Data Availability

The original contributions presented in this study are included in the article. Further inquiries can be directed to the corresponding authors.
